# Drug-induced gingival overgrowth in renal transplants patients

**DOI:** 10.1515/med-2025-1348

**Published:** 2026-03-12

**Authors:** Sarah Monserrat Lomelí-Martínez, Melissa Martínez-Nieto, Ruth Rodríguez-Montaño, Mario Alberto Alarcón-Sánchez, Juan José Varela Hernández, Adrián Fernando Gutiérrez-Maldonado, Juan Carlos Gomez-Mireles, Christian Ramírez Sánchez, Erandis Dheni Torres-Sánchez

**Affiliations:** Department of Medical and Life Sciences, Centro Universitario de la Ciénega, Universidad de Guadalajara, Ocotlán, Mexico; Department of Well-being and Sustainable Development, Centro Universitario del Norte, Universidad de Guadalajara, Colotlán, Mexico; Independent Researcher, Tijuana, BC, Mexico; Department of Health and Illness as an Individual and Collective Process, University Center of Tlajomulco, University of Guadalajara, Tlajomulco de Zúñiga, JAL, Mexico; Department of Integral Dental Clinics, Institute of Research in Dentistry, University Center of Health Sciences, University of Guadalajara, Guadalajara, Mexico; PhD Student in Molecular Biology and Medicine, Molecular Biology Department, University Center of Health Sciences, Universidad de Guadalajara, Guadalajara, JAL, Mexico; Department of Integrated Dentistry Clinics, Centro Universitario de Ciencias de la Salud, Universidad de Guadalajara, Guadalajara, Mexico; Department of Cardiovascular and Thoracic Surgery, Hospital General de Occidente, Zapopan, Mexico

**Keywords:** gingival hyperplasia, sirolimus, tacrolimus, mycophenolate mofetil, cyclosporine A

## Abstract

**Introduction:**

This narrative review describes the scientific evidence on drug-induced gingival overgrowth (DIGO) in kidney transplant patients treated with immunosuppressive agents, particularly Cyclosporine A, focusing on its prevalence, pathogenetic mechanisms, and clinical management strategies.

**Content:**

This study was conducted including PubMed, Scopus, and Web of Science, highlighting clinical studies and case reports.

**Summary:**

DIGO is an oral complication in transplant patients treated with cyclosporine A, and its frequency may increase when combined with calcium channel blockers. However, tacrolimus has shown a lower incidence of DIGO compared with Cyclosporine A, making it a favorable therapeutic alternative in immunosuppressive regimens for renal transplant patients. Mycophenolate mofetil, despite being less directly linked to DIGO, can exacerbate gingival changes when combined with other immunosuppressants by promoting inflammation and connective tissue remodeling. Sirolimus is associated with a lower risk of DIGO compared with calcineurin inhibitors; however, some isolated cases have been reported, particularly in patients previously exposed to Cyclosporine A or when used in combination with calcium channel blockers. Management strategies include proper oral hygiene, dose adjustment or medication substitution, and, in some cases surgical intervention.

**Outlook:**

The fundamental keys to reducing its incidence and severity are a personalized immunosuppressive regimen with a multidisciplinary approach.

## Introduction

Drug-induced gingival overgrowth (DIGO) is a relatively common side effect observed in patients taking certain medications such as anticonvulsants, calcium channel blockers, and immunosuppressants [1], 2]. This condition presents clinically as a fibrous, inflammatory, and painless enlargement of the gingival tissue, generally manifesting in the anterior regions of the maxilla and mandible [1], 2]. This condition exerts a significant impact on patients’ oral health and quality of life [2]. The main relevance of DIGO lies not only in the difficulty of chewing, speaking, and maintaining adequate oral hygiene but also in its significant impact on the patient’s quality of life. This condition of excessive gingival tissue accumulation affects aesthetics and psychosocial well-being and promotes the formation of pseudopockets that create a favorable environment for pathogenic microorganisms [1]. This can trigger periodontal disease, leading to the loss of gingival attachment and, in advanced cases, irreversible damage that results in tooth loss. These cumulative sequelae significantly impair oral function and represent a considerable burden for transplant patients, who are already dealing with complex systemic management [1], [3], [4], [5], [6].

Immunosuppressants are indispensable in the prevention of organ transplant rejection. Several immunosuppressants, including Cyclosporine A (CsA), Tacrolimus (Tc), Mycophenolate mofetil (Mf), and Sirolimus, have been associated with DIGO in kidney transplant patients [7]. The pathogenesis of immunosuppressant-induced DIGO is complex and involves interactions among the drug, pre-existing periodontal inflammation, and genetic predisposition. CsA, a calcineurin inhibitor, has been linked to increased gingival fibroblast proliferation and collagen production, which contribute to gingival tissue overgrowth [2], 7], 8]. Mf inhibits nucleotide biosynthesis by blocking inosine monophosphate dehydrogenase (IMPDH). However, its role in DIGO has remained controversial [2].

This narrative review comprehensively examines the incidence, prevalence, risk factors, underlying mechanisms, and current management strategies for DIGO, with a specific focus on its induction by immunosuppressants such as CsA, Tc, Mf, and sirolimus in renal transplant patients.

## Epidemiology of DIGO

Patients treated with immunosuppressants frequently present with DIGO as a side effect. The prevalence of DIGO varies widely based on factors such as the specific drug used, the population affected, and other risk factors. Available data from the literature reveal that approximately 30 % of patients treated with CsA develop DIGO. However, this prevalence increases to 48–60 % [3] when CsA is combined with nifedipine but decreases to 1.7–3.3 % with amlodipine [9]. Tc is often used as an alternative to CsA for its immunosuppressive properties [1], 7]. These changes may appear within a variable time range, being observed from 4 weeks after starting treatment [1], although in some cases, the onset may take up to 3 months [2]. A possible dose-dependent relationship exists with the immunosuppressive response, particularly CsA, where prolonged exposure and increased serum concentrations are linked to greater severity of gingival changes; however, the exact mechanisms remain to be understood [2], 3], 7]. The onset and severity of DIGO may be influenced by several factors such as gender, age, and pre-existing periodontal conditions. Regarding gender, males are three times more likely to develop DIGO than females; this is probably due to the hormonal effect related to testosterone [1], 7]. Children are at greater risk, particularly when treated with CsA, probably due to heightened fibroblast activity and collagen production in younger individuals [1], 2]. Pre-existing periodontal conditions also play a significant role in the severity and extent of DIGO [1], 7], 8]. The inflammatory response and microorganisms in the dental biofilm associated with these conditions create an environment conducive to excessive periodontal tissue growth in response to immunosuppressant therapy. The concurrent use of adjunctive drugs, particularly calcium channel blockers, e.g., nifedipine, further increases the likelihood of developing DIGO in patients treated with CsA or Tc [1], [7], [8], [9], [10]. In contrast, azathioprine appears to have a protective effect against DIGO in patients placed on CsA or Tc. Although the exact mechanism is unknown, it has been suggested that azathioprine is less effective than other immunosuppressants at inducing fibroblast proliferation and extracellular matrix accumulation [2], 11]. The role of genetic predisposition in DIGO remains a topic of debate. Genetic variations such as polymorphisms in cytochrome P450, HLA, and MDR1 genes, may contribute to susceptibility to DIGO [1], 7]. However, no association was found between DIGO and the interleukin-6 (IL-6) gene polymorphism (−174 G/C) in renal transplant recipients [12].

## Immunosuppressants

As their name implies, immunosuppressant medications are drugs designed to inhibit various functions of the immune system. These agents are primarily used in three key areas of medical treatment. First, they are essential for preventing rejection following organ transplants by suppressing the recipient’s immune response against the transplanted organ [13]. Second, immunosuppressants are used for treating chronic inflammatory diseases: reducing immune activity alleviates the symptoms of these diseases. Third, immunosuppressant drugs play a vital role in managing autoimmune disorders in which the immune system mistakenly attacks the body’s own tissues [14]. These medications are crucial for controlling transplant rejection, and they have broad applications in the treatment of autoimmune diseases such as glomerulonephritis, myasthenia gravis, rheumatoid arthritis, lupus, and Crohn’s disease. Immunosuppressants exert their effects by blocking cytokine production or action, inhibiting DNA synthesis, or suppressing the activation and maturation of immune cells [15]. The frequently prescribed immunosuppressive drugs are CsA, Tc, cyclophosphamide, azathioprine, Mf, sirolimus, methotrexate, glucocorticoids, and leflunomide. These medications may be used singly or in combination, depending on the specific treatment requirements [15]. However, they are often associated with undesirable side effects, e.g., changes in oral tissues [7]. In particular, CsA, sirolimus, and Tc have been linked to gingival overgrowth [13].

### Cyclosporine A (CsA)

**Figure 1:**
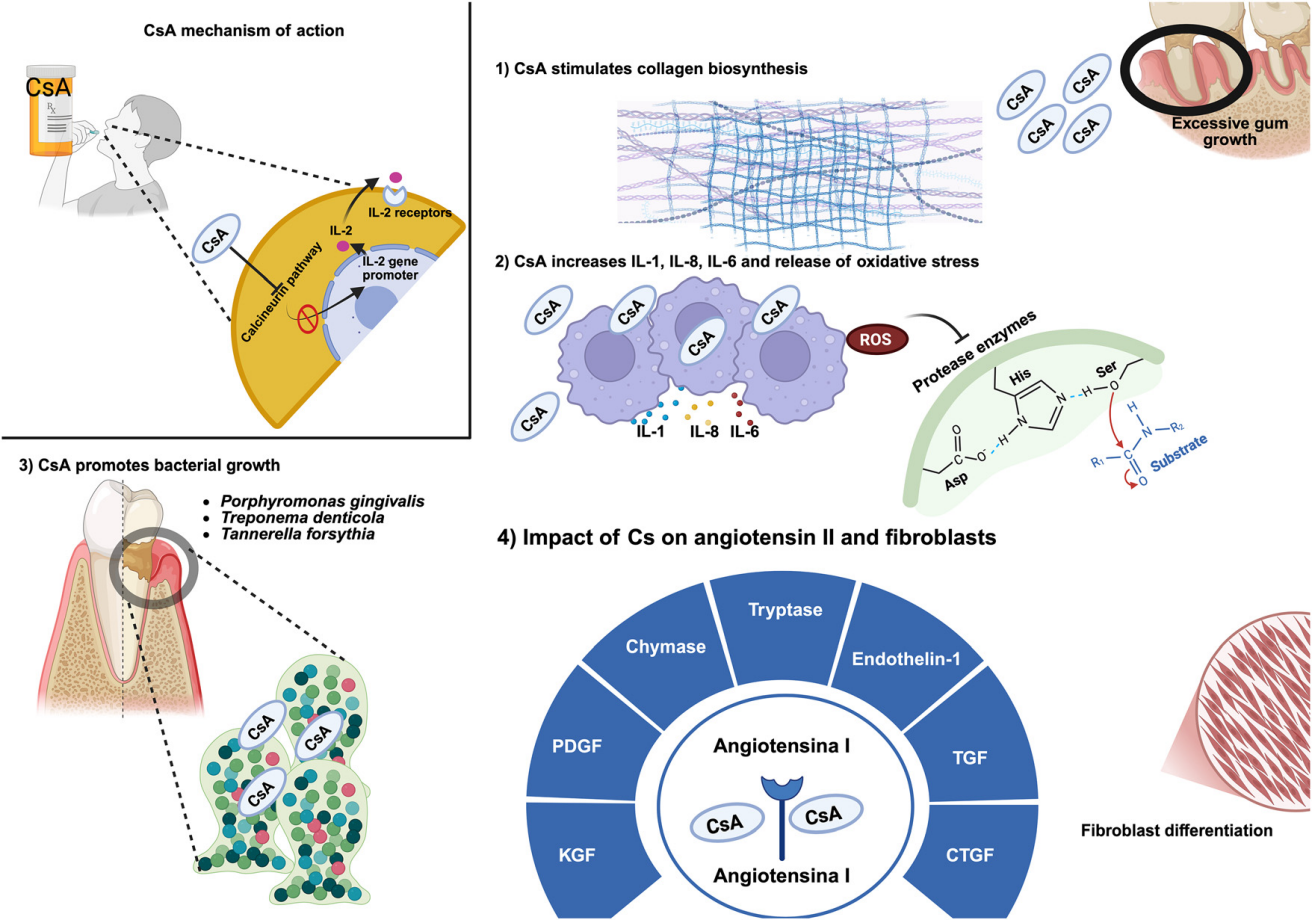
Mechanisms through which CsA affects gingival overgrowth. CsA: Cyclosporine A; IL-2: interleukin-2; IL-1: interleukin-1; IL-6: interleukin-6; IL-8: interleukin-8; ROS: reactive oxygen species; KGF: keratinocyte growth factor; PDGF: platelet-derived growth factor; TGF: transforming growth factor; CTGF: connective tissue growth factor). Created in BioRender. Torres, D. (2024). https://BioRender.com/b64b439.

CsA exerts its effects by inhibiting T-cell production of interleukin-2 (IL-2) through its interaction with cyclophilin, leading to a reduction in immune responses. Additionally, CsA influences collagen biosynthesis and deposition [2]. One of the most well-known adverse effects of CsA is its tendency to induce gingival overgrowth. This condition primarily affects the interdental papillae, resulting in significant damage, although the exact mechanism underlying this side effect remains unclear. Evidence suggests a relationship between CsA-induced gingival overgrowth and increases in salivary levels of pro-inflammatory cytokines, i.e., interleukin-1 (IL-1), interleukin-8 (IL-8), and IL-6 [16], [17], [18] (Figure 1).

Besides its impact on gingival tissues, CsA interferes with periodontal health by creating an environment that complicates plaque removal, a situation that indirectly fosters the progression of periodontal disease [14]. For instance, patients with gingival overgrowth exhibit a higher prevalence of pathogenic bacteria such as *Porphyromonas gingivalis* (*P. gingivalis*), *Treponema denticola*, and *Tannerella forsythia*. The CsA-induced gingival overgrowth favors the formation of pseudo pockets which facilitate the accumulation of dental plaque and CsA residues within them (Figure 1). These pseudo pockets act as bacterial reservoirs, thereby exacerbating gum tissue damage. Additionally, the pharmacologically-induced reduction in the host’s immune response worsens the situation by further promoting periodontal disease progression [16], [17], [18].

CsA modulates the local expression of the renin-angiotensin system, thereby altering levels of angiotensin II and its receptors in gingival tissues. This contributes to gingival overgrowth through the induction of fibroblast differentiation and activation of fibrogenic proteins such as keratinocyte growth factor (KGF), transforming growth factor (TGF), connective tissue growth factor (CTGF), and platelet-derived growth factor (PDGF). The enzymes chymase and tryptase, along with endothelin-1, also play roles in the activation of gingival fibroblasts. Specifically, KGF, CTGF, and TGF drive the progression of CsA-induced gingival overgrowth [7], 17], 18] (Figure 1). It is worth noting that it has been demonstrated that patients treated with CsA exhibit increased overexpression of KGF mRNA, which may explain the extent of gingival overgrowth seen in these patients [19]. Moreover, CsA induces the generation of free radicals that inhibit the protease enzymes responsible for tissue degradation, thereby protecting and enhancing the structural integrity of the extracellular matrix. Furthermore, CsA contributes to gingival overgrowth by increasing the activity of transglutaminase type 2 (TGM-2) in gingival tissue [20] (Figure 1). Interestingly, serum CsA levels below 200 μg/L do not affect gingival overgrowth, whereas concentrations between 400 and 800 μg/L activate fibroblast proliferation [18]. While CsA remains a cornerstone of immunosuppressive therapy, particularly in transplant medicine, its side effects, especially gingival overgrowth, necessitate careful management due to the complications they pose for oral hygiene and periodontal health. Despite its adverse effects such as nephrotoxicity, hepatotoxicity, hypertension, and gingival overgrowth, CsA has revolutionized transplant outcomes by significantly improving graft survival and patient quality of life [14], 21].

### Tacrolimus (Tc)

**Figure 2:**
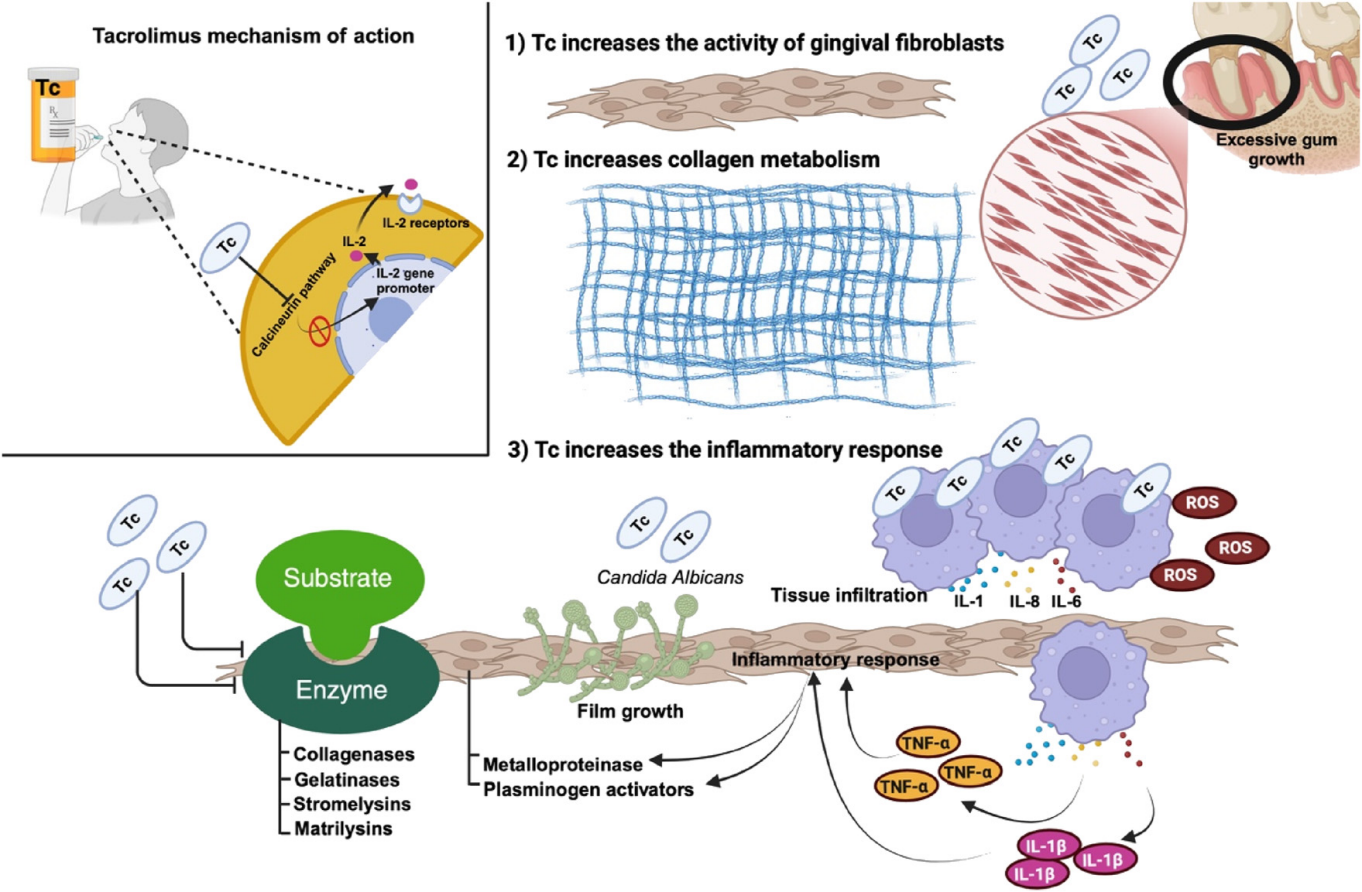
Mechanisms through which Tc affects gingival overgrowth. Tc: Tacrolimus; IL-2: interleukin-2; IL-1, interleukin-1; IL-6: interleukin-6; IL-8: interleukin-8; ROS: reactive oxygen species; TNF-α: tumor necrosis factor alpha; IL-β: interleukin-1 beta. Created in BioRender. Torres, D. (2024) https://BioRender.com/g98f868.

While CsA is highly effective in preventing organ rejection, its significant side effects (gingival overgrowth and related oral health issues), pose considerable challenges. Another alternative is Tc; this is a macrolide molecule and antibiotic, and it shares a similar mechanism of action with CsA, but it leads to decreased hepatic and renal toxicity [13], 14], 21]. Like CsA, Tc functions as a calcineurin inhibitor, thereby playing a pivotal role in immunosuppression by preventing organ rejection following transplantation. Tc achieves this by binding to immunophilin to form a protein complex that inhibits the activity of calcineurin. This inhibition blocks the production of IL-2 and related cytokines which are essential for T-cell activation and proliferation (Figure 2) [22].

Tc is a more potent immunosuppressive agent than CsA, and it has become the preferred treatment for preventing organ transplant rejection. Despite its advantages, Tc is not without side effects such as gastrointestinal disturbances, nephrotoxicity, hypertension, and metabolic abnormalities [14]. Although it shares nephrotoxicity with CsA, Tc induces less severe hypertension and hypertrichosis. In addition, Tc is associated with a lower incidence of gingival overgrowth, especially when is co-administered with calcium channel blockers such as dihydropyridine derivatives or CsA [21]. Shultz et al. indicate that Tc is associated with a lower incidence of gingival overgrowth than CsA [23]. This is consistent with the data published by McKaig et al., where 63 % of the transplanted population that received CsA developed gingival overgrowth while the group that received Tc did not present this condition [24]. However, in renal transplant patients with poor oral hygiene and high plaque indices, gingival overgrowth has been observed in those treated with Tc [25].

The exact mechanism through which Tc contributes to gingival overgrowth remains unclear, although the duration of treatment appears to be a critical factor. For instance, murine models showed that when administered for less than 120 days, Tc did not induce gingival overgrowth, whereas treatment exceeding 180 days resulted in gingival overgrowth [26], 27]. Indeed, evidence suggests that Tc influences gingival fibroblast activity, collagen metabolism, and inflammatory pathways, which may contribute to tissue overgrowth [26]. However, the conflicting findings regarding the influence of Tc on alveolar bone formation, specifically its association with increased bone resorption, may reflect the influence of confounding variables unrelated to Tc treatment [24], 25], 27], 28] (Figure 2). A six-month murine study of Tc treatment revealed epithelial damage to the lingual papillae and mucosa, inflammatory cell infiltration, biofilm formation by *Candida albicans* leading to systemic issues, and reduced enzymatic activities of collagenases, gelatinases, stromelysins, and matrilysins, all of which contributed to gingival overgrowth. Paradoxically, tissue degradation was observed in the tongue. The inflammatory response triggered by Tc was associated with elevated levels of IL-1β and TNF-α, which impacted both epithelial and connective tissues through altered activities of metalloproteinase and plasminogen activator. Additionally, Tc, by impairing antioxidant capacity, may exacerbate gingival overgrowth [29] (Figure 2). Park et al. have emphasized the importance of dosage and timing when evaluating the morphological changes induced by Tc. Depending on the dose, Tc may elicit either apoptotic or proliferative mechanisms. For example, concentrations of 2.4 × 10^−7^ and 2.4 × 10^−6^ M have been shown to enhance osteogenic differentiation by increasing the expression of osteogenic markers in mRNA and promoting cell proliferation [30]. These Tc concentrations are linked to enhanced osteogenic differentiation and increased calcium deposition. The results described above are supported by RNA sequencing studies [31], [32], [33].

### Mycophenolate (Mf)

**Figure 3:**
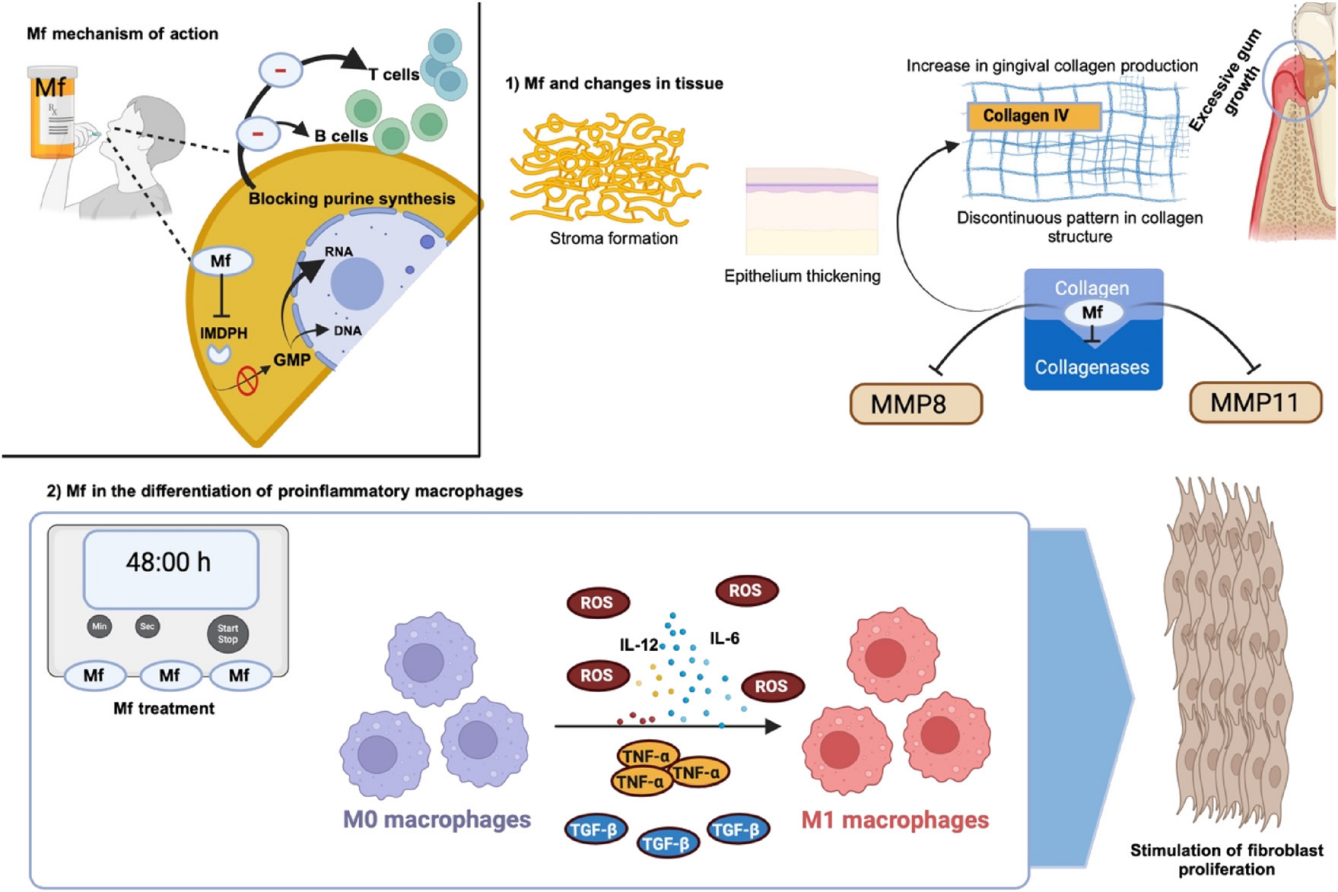
Mechanisms through which Mf affects gingival overgrowth. Mf: mycophenolate; IL-2: interleukin-2; IL-6: interleukin-6; ROS: reactive oxygen species; TNF-α: tumor necrosis factor alpha; IMDPH: inosine-5′-monophosphate dehydrogenase; TGF-β: transforming growth factor-β; MMP8: matrix metalloproteinase-8; MMP11: matrix metalloproteinase 11). Created in BioRender. Torres, D. (2024) https://BioRender.com/f88n085.

Mf has gained prominence in immunosuppressive regimens due to its effective action and relatively low toxicity profile. After it is metabolized to its active form, mycophenolic acid, the drug inhibits IMPDH, an enzyme critical in the biosynthesis of guanosine and deoxyguanosine. This inhibition selectively reduces the proliferation of B- and T-lymphocytes, as these immune cells rely exclusively on the *de novo* pathway of purine nucleotide biosynthesis for their functions (Figure 3). In contrast, other cell types compensate through the use of the salvage pathway of purine biosynthesis, which limits systemic effects [2], 14], 22]. This targeted mechanism makes Mf particularly effective in preventing both acute and chronic organ rejections, thereby cementing its crucial role as a cornerstone in immunosuppressive therapy [2], 14].

Gingival overgrowth could occur as a side effect of immunosuppressive drugs, for example, Mf, particularly in the presence of poor oral hygiene. However, definitive evidence directly linking Mf to gingival overgrowth remains inconclusive. Unlike calcineurin inhibitors such as CsA, the distinct mechanism of action of Mf, i.e., blocking purine synthesis, makes it less likely to induce this side effect. Nevertheless, reports of gingival changes in patients using Mf warrant further investigations, in order to clarify this potential association [14]. Although there are no stand-alone reports on the effect of Mf on gingival overgrowth, its combination with Tc may have a synergistic impact. In transplant patients, this combination has been linked to increased gingival collagen production, resulting in thickened connective tissue stroma and epithelium. These changes are accompanied by a discontinuous expression pattern of type IV collagen, reduced levels of laminin 5, exacerbated bacterial growth, and heightened inflammation [22], 34]. Mf may alter collagenase activity, thereby enhancing the deposition of type IV collagen in gingival tissues and affecting matrix metalloproteinases (MMP8 and MMP11) [35]. This phenomenon was further investigated and it was demonstrated that Mf treatment differentiated M0 macrophages into pro-inflammatory M1 macrophages. This differentiation led to the overexpression of genes such as IDO1, CCL5, and CXCL10, which are associated with the release of inflammatory cytokines (e.g., IL-6, IL-12, and TNF-α), oxidative stress, antimicrobial peptide production, and TGF-β secretion. It is known that these factors collectively stimulate fibroblast proliferation and extracellular matrix growth [2], 36]. Interestingly, a protective effect has been reported for the combination of Mf and CsA due to reduced prevalence of gingival overgrowth [37]. This protective effect may be associated with the interaction triggered by Mf-CsA co-therapy. The IMPDH enzyme’s inhibitory response is particularly important here, as it leads to the inhibition of purine synthesis [2], 14], 22], although there is a lack of conclusive data in the literature regarding this. Thus, these conflicting findings highlight the need for further research to evaluate the precise role of Mf in gingival overgrowth and its interactions with other immunosuppressive agents.

### Sirolimus

In the early stages of transplantation, nephrologists frequently rely on immunosuppressive therapies, particularly calcineurin inhibitors, to prevent organ rejection. However, over time, there has been a shift toward combination therapies aimed at reducing the side effects associated with these treatments. This shift often included sirolimus, which has demonstrated improved long-term graft survival and fewer adverse effects, when compared to traditional regimens [38].

**Figure 4:**
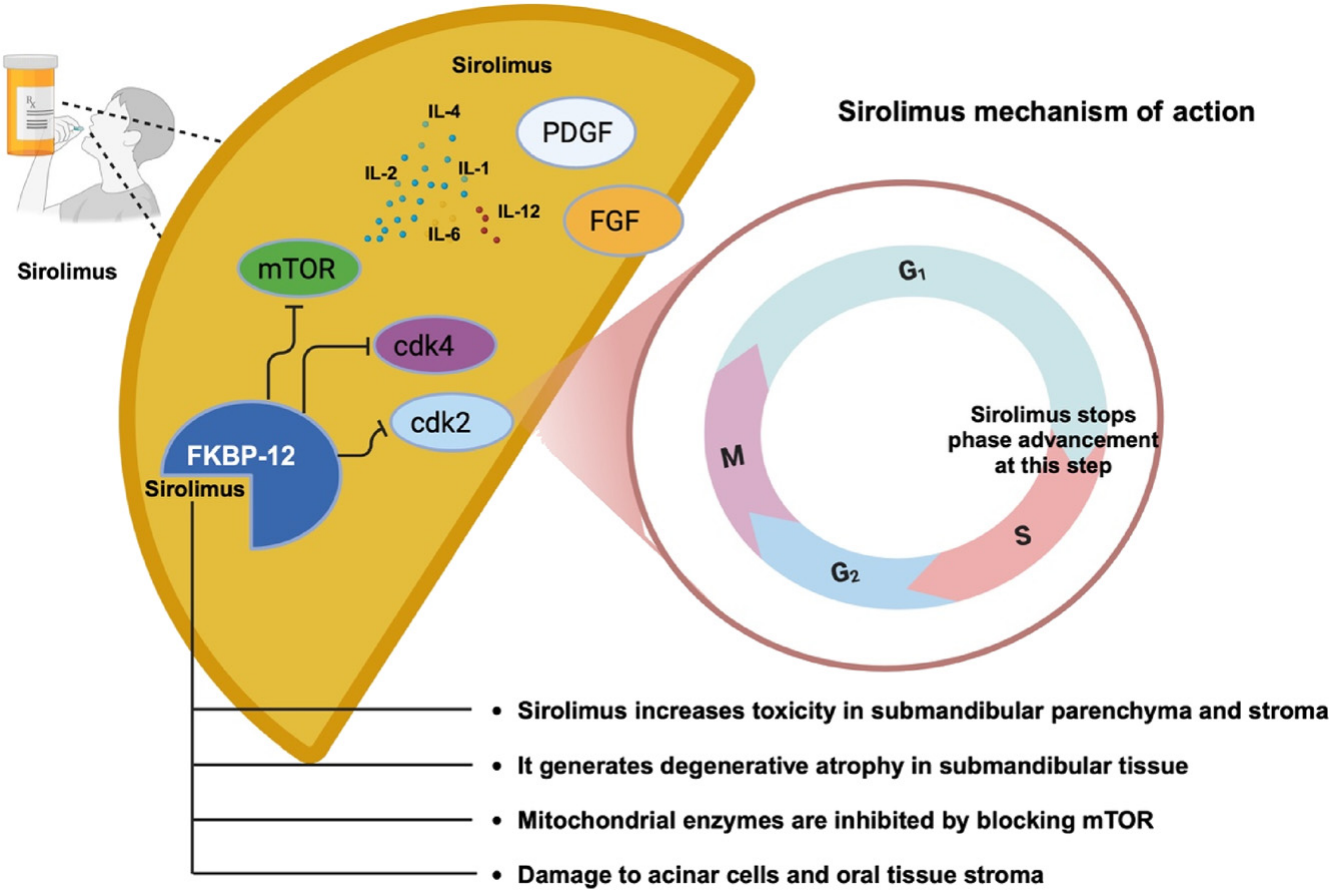
Effect of Sirolimus on gingival overgrowth. IL-1: interleukin-1; IL-2: interleukin-2; IL-4: interleukin-4; IL-6: interleukin-6; IL-12: interleukin-12; mTOR: mammalian target of rapamycin; cdk4: cyclin-dependent kinase 4; cdk2 cyclin-dependent kinase 2; PDGF: platelet-derived growth factor; FGF: fibroblast growth factors. Created in BioRender. Torres, D. (2024) htsRender.com/n70y449.

It differs from calcineurin inhibitors in its mechanism of action and side effect profile. Sirolimus is a rapamycin inhibitor, and it plays a crucial role in modulation of cellular activation by influencing growth factors. The drug binds to the cytosolic receptor FK-binding protein 12 (FKBP-12). This inhibits cellular proliferation during the G1 to S phases of the cell cycle. This inhibition occurs through the blocking of the mammalian target of rapamycin (mTOR) pathway, thereby affecting the production of PDGF, fibroblast growth factor (FGF), and interleukins (IL-1, IL-2, IL-3, IL-4, IL-6, and IL-12). Additionally, sirolimus inhibits cyclin-dependent kinase complexes (cdk4/cyclin D and cdk2/cyclin E) which are essential for progression from the G1 phase to the S phase (Figure 4). This inhibition prevents the proliferation of T and B lymphocytes during this critical phase of the cell cycle [39], [40], [41]. Unlike CsA and Tc which are strongly associated with gingival overgrowth, sirolimus is thought to present a lower risk of inducing this enlargement. Indeed, sirolimus is linked to fewer cases of gingival overgrowth, when compared to the more commonly used immunosuppressants [42].

Although sirolimus is less likely to cause gingival overgrowth than calcineurin inhibitors, the risk is not entirely absent, particularly when it is used in combination therapies. For instance, when sirolimus is administered along with MF, there is a potential for gingival overgrowth, although the incidence remains lower when compared to regimens that heavily rely on calcineurin inhibitors [43]. Cases of gingival overgrowth under sirolimus-based therapy have been documented, but they are typically mild and fall below significant clinical thresholds [21]. A 2008 cross-sectional study of kidney transplant patients receiving sirolimus revealed that 20.8 % of these patients developed gingival overgrowth. In particular, patients who were also treated with calcium channel blockers in addition to sirolimus, experienced more severe gingival overgrowth than those who received sirolimus alone. The authors of this study suggested that previous exposure to CsA and Tc may contribute to the development of gingival overgrowth in patients receiving sirolimus [44]. In addition to gingival changes, sirolimus treatment has been associated with mouth ulcers, hypogeusia, and xerostomia, especially when high doses are used. These effects are believed to exacerbate oral infections and promote bacterial proliferation, most likely due to the inhibition of the mTOR pathway [45], 46]. In a murine model, long-term treatment with calcineurin inhibitors followed by sirolimus (10 mg/kg/day) produced significant morphological changes in the salivary glands. The transition to sirolimus treatment led to a decrease in collagen deposition and degeneration of both parenchymal and stromal elements. Interestingly, the treatment also resulted in cytoplasmic vacuolization of ductal and acinar cells, dissociation of collagen fibers, fibroblast degeneration, and dilation of blood vessels [47]. Sirolimus causes marked toxicity to the parenchyma and submandibular stroma, leading to oral alterations [48]. Specifically, in a murine model, sirolimus exposure caused degenerative atrophy in submandibular tissue, probably due to the inhibition of mitochondrial oxidative phosphorylation enzymes. This led to damage of acinar cells and the stromal tissue of the oral cavity. The treatment also resulted in dissociation of collagen fibers, degeneration of fibroblasts, and dilation of blood vessels [41]. These findings are consistent with those reported by Grabowska and colleagues [47]. Although sirolimus is generally associated with a lower risk of gingival overgrowth compared with Tc and CsA, it may still contribute to its development, particularly when used in combination with other immunosuppressive agents; therefore, close clinical monitoring is recommended [13].

## Immuno-cellular and molecular effects on gingival overgrowth

DIGO is a disease related to different drugs, mainly calcium channel blockers, anticonvulsants, and immunosuppressants. Gingival growth is highly related to cellular and molecular immunological processes. These processes involve the expression of various cytokines such as TGF-β1 and other proteins such as collagen [49], 50]. Fibroblasts increase collagen expression, promoting fibrosis of the gingival tissue. This finding confirms the relevance of fibroblasts in this disease [51], 52]. CsA is the drug most associated with gingival growth [53], 54]. In addition, CsA is known to reduce or evade the process of programmed cell death (apoptosis) of fibroblasts, favoring their survival and contributing to the progressive accumulation of gingival tissue [55]. This antiapoptotic action is considered a fundamental mechanism in the development of DIGO. Furthermore, it is known that CsA manages to reduce or evade the process of programmed cell death (apoptosis) of fibroblasts, which allows for cell survival and, therefore, gingival tissue [55]. Plaque accumulation can be increased due to the same gingival growth that provides greater structure to support the plaque, especially where a pseudopocket is formed [56]. Once gingival inflammation begins due to plaque accumulation, the expression of IL- 1βincreases, and this cytokine, together with CsA, enhances the expression of IL-6, another key proinflammatory cytokine in the production of collagen by gingival fibroblasts [57], [58], [59]. The effect of other cytokines such as vascular endothelial growth factor (VEGF) and FGF-2 has also been observed, as they can stimulate the proliferation of fibroblasts [60]. Likewise, the effect of these medications can promote the mitogenic activity of both gum epithelial cells and fibroblasts because they express proliferation markers such as Ki67 and cyclin B1 [57], 61].

On the other hand, an effect has also been observed for medications that block calcium channels such as nifedipine and amlodipine. These medications promote the accumulation of calcium citrate within cells such as fibroblasts, triggering the proliferation of these cells [58], 61]. Likewise, it has been seen that nifedipine stimulates the production of IL-6 and IL-8 [62]. This evaluated expression of proinflammatory cytokines, as well as excess extracellular matrix formed mainly by type I collagen, is the main characteristic of DIGO [7]. Despite the evidence regarding the effect of pharmacological treatments on gingival growth, it is important to consider the influence of other factors such as heredity, age, immunodeficiency problems, etc., since not all subjects who consume these drugs show gingival growth [51], 52], 63].

An interesting aspect of the pathophysiology of DIGO lies in the nature of the proliferative tissue. Phenytoin is frequently associated with a fibrotic condition, characterized by a high density of collagen fibers and minimal inflammatory infiltration. In contrast, CsA typically presents with predominantly inflammatory lesions and limited fibrosis, while nifedipine exhibits a mixed pattern, combining both inflammatory and fibrotic components. These findings not only highlight histopathological diversity but also carry important clinical implications for treatment outcomes, therapeutic strategies, and prognosis [64].

Additionally, greater expression of CTGF is observed in patients treated with phenytoin compared to CsA or nifedipine [64]. The low fibrosis and increased inflammation by CsA demonstrate not all DIGO is merely fibrous [65], [66], [67]. Like CsA, phenytoin-induced growth is mediated by the expression of TGF-β and CTGF, generating greater extracellular matrix production in addition to inhibiting cell apoptosis [64], 68]. Another study discovered that periostin, a fibrosis marker, is upregulated in nifedipine-induced gingival overgrowth. Nifedipine enhances TGF-β signaling, leading to increased periostin expression [69]. Periostin, like CTGF, is a matricellular protein that contributes to fibrosis. Periostin has been found to promote extracellular matrix production through its interactions with the procollagen-C-proteinase, bone morphogenetic protein 1 (BMP1), which transforms numerous critical extracellular matrix protein precursors into mature functioning extracellular matrix structures [70].

## Clinical picture

DIGO is a condition that manifests as a painless growth of gingiva; it involves the marginal gingiva and the attached gingiva as a globular, thickened pink mass. Despite its characteristic hard-elastic consistency, it retains a normal texture, without inflammation or tendency to bleed [1], [3], [4], [5], [6, 71]. However, as the lesion evolves, it becomes inflamed with red or red-blue discolorations, in addition to bleeding. It spreads horizontally and vertically [1]. Frequently, DIGO affects the anterior vestibular surfaces, particularly the incisors and canines [1], 3]. In advanced forms of DIGO, the enlargement of the gingiva negatively impacts mastication, speech, and aesthetics. Additionally, it interferes with oral hygiene, leading to an increased susceptibility to the development of other oral lesions [1], 3], 71].

## Treatment of DIGO in renal transplant patients

The management of DIGO depends on the specific characteristics of each patient. However, different treatment phases have been successfully implemented for patients with kidney transplants who are taking immunosuppressants [72], 73]. The systemic phase involves collecting information on concomitant pathologies and the medications prescribed for them, followed by consultation with the physicians involved in the treatment. The primary option for treating DIGO is to adjust the dose of medication or to replace the medication entirely; a decision that must be made by the medical team (Table 1). Some studies have found a decrease in DIGO when CsA is replaced with Tc [11], 74]. In contrast, Cañas et al. did not report any changes in the clinical condition of the patient they studied. In this case, after taking Tc for some time, the patient developed DIGO again [72]. This outcome may be attributed to prior treatment with CsA, which increases gingival overgrowth in patients taking Tc, thereby making it a potential residual effect of CsA. The hygiene phase is the cornerstone of the treatment for DIGO. The main focus of this phase is to control bacterial plaque through various measures in order to reduce gingival inflammation and create a healthier oral environment. This phase has several components such as oral hygiene instructions, motivation and support, scaling and root planning, mouthwashes, and dental biofilm control. Oral hygiene instructions involve teaching patients the correct brushing techniques, use of dental floss, and the implementation of interdental brushes. It has been stressed that patients and caregivers should be educated about the importance of meticulous oral hygiene, which is crucial in arresting the development and recurrence of gingival overgrowth [3], 4], 6], 16], [71], [72], [73, 75], 76]. Motivating patients to adhere to good oral hygiene and providing ongoing support are essential to ensure long-term compliance. For patients with special needs, mental disabilities, or difficulties in maintaining adequate oral hygiene, additional strategies may entail using electric toothbrushes and dental flossing devices. Scaling and root planning involve the professional removal of plaque and tartar (dental calculus) from above and below the gum line. This helps reduce gingival inflammation while creating a smoother surface for the gingiva to heal properly [3], 6], 16], [71], [72], [73]. An antiseptic mouthwash such as 0.12 % chlorhexidine, may be incorporated to help control plaque and reduce gingival inflammation [3], 71], 73], 77]. Plaque control is maintained through regular follow-ups to assess the effectiveness of oral hygiene measures and make adjustments as necessary. It is essential that patients achieve satisfactory plaque control before considering surgical therapy, as plaque accumulation increases the risk of DIGO recurrence [72]. The hygiene phase offers several benefits: reduction in gingival inflammation, plaque control, improved periodontal health, preparation for surgical therapy, and prevention of recurrence. When DIGO is severe, unresponsive to non-surgical treatment, or when it interferes with oral function, the surgical phase may be considered, with the goal of removing excess gingival tissue. Consideration for surgery is based on several factors such as severity of the enlargement, underlying periodontal diseases, and the general health of the patient. The surgical techniques that may be considered are gingivectomy, gingivoplasty, and flap surgery [3], [71], [72], [73, 75], 76], 78]. The most common surgical technique for the management of DIGO is gingivectomy, which involves the excision of excess gingival tissue and reshaping of the gingiva to create a more physiologic and aesthetic contour [3], [71], [72], [73, 75], 76], 78]. Gingivoplasty is performed to reshape the gingiva and enhance its aesthetic contour; it is commonly done in combination with gingivectomy in order to improve the aesthetic results [73]. Flap surgery is a technique that involves lifting a flap of gingival tissue to access the underlying bone and remove the enlarged tissue. This modality is used in more complex cases of DIGO where access to the bone tissue or deeper periodontal tissues is needed. The surgical phase may be executed with a scalpel [3], 73], 76], electrocautery [79], or laser [4]. The surgical modality effectively removes excess gingival tissue and improves oral function and aesthetics. However, it is essential to consider the underlying factors that contribute to gingival overgrowth, such as control of bacterial plaque and gingival inflammation, in order to prevent recurrence and maintain long-term gingival health. Patients should be integrated into the periodontal support therapy phase which includes a periodontal maintenance phase aimed at preventing recurrence. In this phase, follow-up appointments should be scheduled every three months, during which it is crucial to emphasize the importance of meticulous personalized oral hygiene [72]. Periodic control of bacterial biofilm and oral hygiene instruction are vital for long-term success [4], 72], 75], 77]. Notwithstanding the implementation of the phases of periodontal treatment, DIGO may present a high recurrence rate of up to 34 %, and may manifest within 18 months, even after surgical therapy [16]. The factors that contribute to recurrence are the continued use of the offending medication, poor plaque control, gingival inflammation, and lack of patient adherence to oral hygiene practices [16], 72]. If the medication responsible for DIGO is not discontinued or changed, the risk of recurrence remains high. The continued administration of drugs such as CsA, Tc, and amlodipine, which are frequently used in transplant patients, may lead to recurrence of AG [16], 72]. The accumulation of bacterial plaque is a significant risk factor for the development and recurrence of DIGO. Furthermore, the complexity of maintaining adequate oral hygiene due to DIGO itself exacerbates the problem [16], 72]. Gingival inflammation, whether pre-existing or persistent, significantly increases the likelihood of recurrence. This inflammation may be associated with the presence of bacterial plaque, tissue response to drugs, or a combination of both [16], 72], 75], 76]. If a patient does not adhere to the recommended oral hygiene practices, or fails to attend maintenance visits, they are more likely to experience a recurrence. It is important to note that this lack of engagement may be attributed to several factors, such as difficulty in accessing dental care, lack of understanding of the importance of oral hygiene, and mental health issues. The treatment of DIGO in kidney transplant patients taking immunosuppressants requires a multidisciplinary approach. Collaboration between the physician, periodontist, and patient is essential for achieving adequate DIGO management. Plaque control is crucial in all cases, and periodontal surgery may be necessary in severe cases. Patient education and periodontal support therapy are vital for preventing recurrence and maintaining long-term periodontal health.

**Table 1:** Clinical cases of DIGO.

Study	General and systemic patient data					Intraoral findings and dental management						
	Gender	Age (years)	Comorbidity	Time since transplant (years)	Systemic treatment	Location of DIGO	Biofilm and/or calculus	Radiographic findings	Diagnosis	Treatment		
										Pharmacological change	Dental Management	Therapeutic Management
Daly 1992 [77]	Female	65	Chronic active hepatitis	No data	CsA 400 mg/day	Lower arch	Dental biofilm Calculus	No data	Clinical	Changed CsA 400 mg/day to 200 mg/day	Oral hygiene instruction Clean around the gum margins using cotton buds soaked in 0.2 % chlorhexidine Overhanging amalgam restorations were smoothed and recontoured	Chlorhexidine oral rinse 0.12 % (10 mL, 2 × per day)
Hernández et al. 2000 [11]	Female	50	No data	7	CsA 4–5 mg/kg day Corticosteroids 10 mg/day Prednisone 10 mg/day Phenytoin 0.1 g/8 h Carbamazepine 200 mg/8 h	Lower arch Upper arch	Dental biofilm Calculus	Bone loss for the lower incisors	Clinical	Change CsA to Tc 7 mgr/day	Oral hygiene instruction Scaling and root planning	Chlorhexidine oral rinse 0.12 %
Bahamodes 2007 [75]	Male	16	Hypertension Alcoholism	4	CsA Nifedipine (Dosis not mentioned)	Lower arch Upper arch	Dental biofilm	No data	Histopathology		Oral hygiene instruction Scaling and root planning Gingivectomy	
Aral et al. 2015 [4]	Male	54	Hypertension	4	CsA 500 mg/day Nifedipine 30 mg/day Warfarin 5 mg/day	Lower arch Upper arch	Dental biofilm	Generalized alveolar bone loss	Histopathology	CsA changed to Tc, Nifedipine changed to captopril 100 mg/day	Oral hygiene instruction Scaling and root planning Diode laser (940 nm)	Prophylactic amoxicillin (2,000 mg single dose) Post-operatory cephalosporin (cefaclor 250 mg/day).
González et al. 2016 [80]	Male	74	Hypertension Gout disease	14	CsA 75 mg/12 h Nifedipine 20 mg/6 h Atenolol 100 mg/day Allopurinol 100 mg/day Prednisone 5 mg/day Colchicine 0.6 mg/day	Lower arch Upper arch	Dental biofilm	Deep marginal bone resorption, almost complete in groups III and VI.	Histopathology		Oral hygiene instruction Periodontal treatment Extraction of teeth with severe periodontal involvement (groups III and VI)	
Kendall et al. 2017 [78]	Male	23	No data	4	Mycophenolate mofetil Tc Amlodipine (Dosis not mentioned)	Lower arch Upper arch	Dental biofilm Calculus	No data	Clinical	Discontinuation of causative medications	Gingivectomy	
Cañas et al. 2017 [72]	Female	22	Hypertension	14	Tc 7 mg/day Mycophenolate sodium 1,080 mg/day Amlodipine 10 mg/day Losartan 150 mg/day Carvedilol 75 mg/day	Lower arch Upper arch	Dental biofilm	Bony ridges of normal height	Histopathology	Changed mycophenolate sodium 1,080 mg/day to 1,040 mg/day	Hygienic phase Gingivectomy	Acetaminophen 1 g/8 h
Malek 2018 [16]	Female	21	No data	5	CsA 125 mg/day Prednisolone 5 mg/day Mycophenolate mofetil 500 mg/day	Lower arch Upper arch	Dental biofilm Calculus	Marginal (coronal third) horizontal alveolar bone loss which was more pronounced at the lower incisors.	Clinical		Oral hygiene instruction Scaling and root planning Extraction of the remaining root of tooth #26.	Amoxicillin plus clavulanic acid 1 g/8 h 2 per days
Chang et al. 2018 [6]	Female	51	Hypertension Gout	16	CsA 50 mg/twice a day Prednisolone 5 mg/day Mycophenolate mofetil 250 mg/twice a day Atenolol 100 mg/day Benzbromarone 50 mg/every other day Colchicine 0.5mg/every other day	Lower arch Upper arch	Dental biofilm Calculus	Alveolar bone loss	Clinical		Oral hygiene instruction Scaling and root planning	Prophylactic antibiotics, amoxicillin, 2 g/before each appointment.
Veitia et al. 2019 [71]	Male	44	Hypertension (10 years)	2	CsA 50 mg/12 h Atenolol 100 mg/day	Lower arch Upper arch	Dental biofilm Calculus	No bone loss	Clinical		Motivation and health education Oral hygiene instruction Scaling and root planning Gingivectomy	Chlorhexidine oral rinse 0.12 %
Nanda et al. 2019 [3]	Male	42	Hypertension	11	Atenolol 100 mg/day	Upper arch Right quadrant	Dental biofilm Calculus	No data	Histopathology		Oral hygiene instruction Scaling and root planning Gingivectomy	Azithromy 500 mg once daily for 5 days starting 2 days before the surgical procedure Chlorhexidine oral rinse 0.12 %
Morales aguiar 2019 [81]	Male	50	Hypertension Bronchial asthma Hepatitis C	4	CsA 0.8 mg/12 h Amlodipine 20 mg/day Prednisone 5 mg/day	Lower arch Upper arch	Dental biofilm Calculus	Mild and moderate generalized bone loss.	Clinical		Oral hygiene instruction with the bass brushing technique Scaling and root planning Gingivectomy	Amoxicillin 500 mg/12 h
Michea 2020 [73]	Male	27	Hypertension Diabetes mellitus	10	CsA 25 mg/12 h Nifedipine 20 mg/12 h Diltiazem 60 mg/day Metformin 850 mg/8 h	Lower arch Upper arch	Dental biofilm Calculus	No data	Clinical	.	Instruction on sweeping brushing technique Scaling and root planning in two sessions Gingivectomy	Amoxicillin 2 gr/1 h prior to all non-surgical and surgical periodontal treatment Chlorhexidine oral rinse 0.12 % for two weeks

CsA, ciclosporina A; Tc, tacrolimus; DIGO, drug-induced gingival overgrowth.

Azithromycin (AZI) is a broad-spectrum antibiotic from the macrolide group of the azalide subclass that suppresses protein synthesis in both Gram-positive and Gram-negative aerobes. It has been suggested that this macrolide exerts an inhibitory effect on CsA-induced gingival overgrowth in *in vitro* [80], [81], [82], animal [83], and human investigations [84], 85]. The *in vitro* study conducted by Kim et al. in 2008 analyzed the effect of AZI on cell proliferation and CsA-induced collagen turnover in gingival fibroblasts from renal transplant patients and healthy subjects. This drug inhibited CsA-induced proliferation of fibroblasts from renal transplant patients, and it elevated the activities of reduced MMP-1 and MMP-2. In CsA-treated renal transplant fibroblasts, AZI blocked total collagen accumulation in the culture media and reversed the increase in the mRNA level of type I collagen [82]. These findings suggest that AZI may be used to improve gingival overgrowth conditions by blocking CsA-induced cell proliferation and collagen synthesis, and by activating MMP-2 in gingival fibroblasts. Ratre and Mehta (2016) evaluated the effect of AZI on gingival overgrowth induced in rats by the combination of CsA and Nifedipine. Thirty male rats were divided into three equal groups: group 1 (control) received olive oil only; group 2 received a combination of CsA and Nifedipine in olive oil throughout the study period, while group 3 received combination therapy of CsA and Nifedipine, with AZI added for one week in the fifth week. Rats in groups 2 and 3 showed significant gingival overgrowth, when compared to group 1 rats. However, in group 3 (AZI), gingival overgrowth was observed up to the fourth week, but a significant decrease in gingival overgrowth was seen during weeks 6–8 after AZI administration in the fifth week [83]. These results indicate that AZI is effective, safe, and cost-effective in treating DIGO in patients receiving combined therapy with CsA and Nifedipine. The authors suggested that AZI should be considered a first-line drug in the treatment of DIGO cases. The clinical trial by Citterio et al. (2001) evaluated the efficacy of AZI treatment for DIGO in 35 kidney transplant patients under CsA-based immunosuppression. The antibiotic was administered on day 1 at an oral dose of 500 mg, and at a dose of 250 mg from day 2 to day 5. The results showed that gingival overgrowth was successfully treated with a 5-day AZI cycle in 86 % of patients, and all of them reported cosmetic satisfaction and the disappearance of bleeding and pain [84]. The authors suggested AZI treatment for gingival overgrowth and recommended managing CsA-induced gingival overgrowth with a 5-day AZI cycle every 8–12 months, in order to prevent recurrence while increasing the success rate of treatment. On the other hand, Tokgöz et al. (2004) demonstrated the usefulness of AZI at a dose of 500 mg/day for 3 consecutive days for treating CsA-induced gingival overgrowth in kidney transplant patients, without the presentation of side effects [85]. Finally, the systematic review and meta-analysis by Teshome et al. (2020) investigated the effect of AZI on CsA-induced gingival overgrowth and concluded that this antibiotic significantly reduced CsA-induced gingival overgrowth in patients. They showed that AZI produced statistically significant reduction in bleeding on probing [86]. It was also revealed that AZI had no significant effect on plaque index and probing depth; this was attributed to the fact that these two clinical parameters demonstrate true periodontal disease in the patients, while gingival overgrowth is a pseudo-enlargement due to the drug that does not present bone loss. Prospects for DIGO research in renal transplant patients receiving immunosuppressants should focus on several key aspects that require further investigation. Studies should emphasize molecular regulation, use of adjuvant therapies, periodontal control, and the personalization of immunosuppressive treatments so as to improve the management of DIGO and mitigate its impact on the oral and general health of patients. Although some underlying mechanisms of immunosuppressant-induced DIGO have been identified, they are not yet fully understood. The regulation of factors such as MMP, CTGF, and cytokines (e.g., IL-6) has been linked to DIGO [87]. Research focused on developing interventions to regulate these factors may prevent gingival overgrowth without compromising the immunosuppressive efficacy of the treatment. It has been suggested that some compounds found in green tea [e.g., epigallocatechin-3-gallate (EGCG)], inhibit CTGF expression and reduce the size of gingival overgrowth. The potential for research on the use of these and other compounds as adjuvant treatments for patients undergoing immunosuppressive therapy is encouraging. The reduction of adverse effects (e.g., DIGO) through personalized immunosuppressive therapies may be of great interest. Adjustments of CsA dose, or substitution with another immunosuppressant such as Tc, has sometimes proven to be a viable alternative [11], 74]. However, the risk of recurrence remains high [16]. Future research should investigate surgical therapies that lower the risk of recurrence and enhance the quality of life of the patient.

## Conclusion and future prospects

DIGO, particularly in renal transplant patients undergoing immunosuppressive treatment, is a clinical phenomenon of great importance for both medical and dental practices. Throughout this manuscript, we have described the underlying pathogenetic mechanisms, risk factors, and the most current therapeutic management options. The drug CsA continues to be the immunosuppressant most associated with DIGO. However, an alternative drug such as Tc has produced promising results in minimizing this adverse effect. Managing this condition requires a multidisciplinary approach that integrates collaboration between the medical team and periodontal specialists through the employment of personalized strategies based on the evolution and clinical severity of each patient. Implementing rigorous hygienic measures and, in the most severe cases, surgical interventions with different modalities are fundamental strategies for enhancing the patient’s quality of life and reducing the recurrence of DIGO. In the future, it is expected that newer research perspectives will focus on the molecular regulation as well as the optimization of adjuvant therapies, which will allow for more effective management of this complication without compromising the success of immunosuppressive treatment. The development of targeted therapies and the personalization of immunosuppressive regimens are key areas that merit further research in order to minimize adverse effects and improve the overall prognosis of patients.
